# Evaluation of the Efficacy of the Traditional Chinese Medicine Formulation Ru-Yi-Jin-Huang-Saan on Colles Fracture After Surgery: Protocol for a Randomized, Double-Blind, Placebo-Controlled Trial

**DOI:** 10.2196/56849

**Published:** 2025-03-05

**Authors:** Lien-Cheng Lin, Wei-Hsun Wang, Wei-Kai Chang, Jyun-Liang Gao, Ru-Chang Yang, Po-Chi Hsu, Lun-Chien Lo

**Affiliations:** 1 Graduate Institute of Chinese Medicine China Medical University Taichung Taiwan; 2 Department of Traditional Chinese Medicine Changhua Christian Hospital Changhua Taiwan; 3 Department of Orthopedic Surgery Changhua Christian Hospital Changhua Taiwan; 4 Department of Post-Baccalaureate Medicine College of Medicine National Chung Hsing University Taichung Taiwan; 5 School of Chinese Medicine China Medical University Taichung Taiwan; 6 Department of Chinese Medicine China Medical University Hospital Taichung Taiwan

**Keywords:** traditional Chinese medicine, Ru-Yi-Jin-Huang-Saan, external application, Colles fracture, Patient-Rated Wrist Evaluation, PRWE, PRWE score, surgeries, fracture, randomized controlled trial, RCT, alternative treatment, postoperative, protocol, Western medicine, wrist evaluation, pain relief medication

## Abstract

**Background:**

Colles fracture, a common wrist injury, often requires surgical intervention. After surgery, patients may experience persistent pain and reduced wrist function, potentially resulting in long-term disability. In clinical practice, traditional Chinese medicine practitioners frequently use Ru-Yi-Jin-Huang-Saan (RYJHS) to treat such patients in Taiwan. RYJHS is a traditional Chinese herbal formula with a history spanning centuries, primarily used topically for the treatment of bone fractures and the promotion of healing. However, there is currently a lack of substantial clinical evidence supporting its efficacy in the management of postsurgical Colles fractures. To the best of our knowledge, there are no studies evaluating the clinical effectiveness of RYJHS.

**Objective:**

This study aims to investigate the therapeutic potential of RYJHS in postsurgical Colles fracture cases. An additional objective is to provide an alternative treatment option for postoperative patients unable to take anti-inflammatory and pain relief medications.

**Methods:**

This is a protocol for a randomized, double-blind, placebo-controlled trial. A total of 100 postoperative patients with Colles fracture, aged 20-80 years, will be recruited for this study. They will be randomly assigned to either the experimental or control group in a 1:1 allocation ratio. Both groups will receive standard postoperative Colles fracture treatment. The primary outcome measure will assess wrist functional recovery using the Patient-Rated Wrist Evaluation score. Secondary outcomes will include C-reactive protein levels and ultrasound measurements of wrist swelling. All of these examinations will be assessed at baseline, 3 days after surgery, and 6 days after surgery. In addition, the Dyshidrotic Eczema Area and Severity Index will be used to monitor for adverse skin reactions.

**Results:**

This protocol was registered at ClinicalTrials.gov on December 6, 2022. It was performed in accordance with the approved guidelines and regulations of the participating institutions. Recruitment began in May 2023, with data collection expected to conclude in May 2025. Study completion is expected in December 2025.

**Conclusions:**

This is the first protocol discussing the assessment of the therapeutic efficacy and safety of topical traditional Chinese medicine in patients after fracture surgery. The protocol will establish an integrated care model combining both traditional Chinese medicine and Western medicine for postsurgical fracture cases.

**Trial Registration:**

ClinicalTrials.gov NCT05638360; https://clinicaltrials.gov/ct2/show/NCT05638360

**International Registered Report Identifier (IRRID):**

DERR1-10.2196/56849

## Introduction

Distal radial fracture is the most common fracture clinically and is approximately one-sixth of cases in emergency departments in the United States [1]. Studies on the Taiwanese population have shown that the prevalence of Colles fracture was 10.2-14.5 per 10,000 people [2]. This situation causes huge losses to the social economy, as well as decreased school attendance, lost work hours, care needs, and permanent disability [3]. In the Western world, closed reduction and cast immobilization are the first choices of treatments in most cases of distal radius fracture [4]. However, surgery intervention is the first choice in Taiwan. Unfortunately, pain and swelling after surgery may hinder rehabilitation and the regaining of hand function, such as postponed recovery of range of motion, daily function, and muscle power [5]. To control pain and swelling, physicians often use ice packing [6], opioids, nonsteroidal anti-inflammatory drugs (NSAIDs), and steroids [7-9]. Some studies reported that these drugs can have the risk of addiction, lead to respiratory restriction, delay fracture wound healing, raise the risk of osteoporosis, and raise glucose levels [10-13]. Since internal medicine has side effects, external medicine should be used to reduce swelling and relieve pain after the operation.

Traditional Chinese medicine (TCM) has been used to treat fractures for thousands of years. In animal studies, TCM extracts have been shown to accelerate bone healing and prevent delayed fracture healing and nonunion [14]. Other studies have also shown that TCM inhibits the inflammatory response in osteoarthritis rat models [15,16]. Until now, there has been no published study on the application of TCM in the treatment of postoperative fractures. However, TCM for external application has been widely used to treat swelling and pain after fracture. Therefore, we have designed an experiment to verify the curative effect of the external application of TCM in fracture surgery.

Ru-Yi-Jin-Huang-Saan (RYJHS) is a TCM herbal patch composed of a fixed blend of TCM ingredients combined with water. It is traditionally applied to relieve swelling and pain in the early stages of musculoskeletal injuries, attributed in TCM theory to its heat-clearing and swelling-reducing properties. Modern pharmacological studies also confirm its antibacterial, anti-inflammatory, wound-healing, and hemostatic effects [17,18]. As recorded in the classic TCM text, *The Golden Mirror of Medicine*, RYJHS is prescribed for conditions such as furuncles, carbuncles, traumatic wounds, mumps, contact dermatitis, lower limb edema, mastitis, and cellulitis. An animal study also demonstrated that RYJHS significantly accelerated fracture healing, notably enhancing collagen formation and bone cell metabolism [19].

The primary components of RYJHS include *Trichosanthis radix, Rhei radix et Rhizoma, Phellodendri cortex, Curcumae longae rhizoma, Angelicae dahuricae radix, Magnoliae cortex, Glycyrrhizae radix et Rhizoma, Citri reticulatae pericarpium vetum, Atractylodis rhizoma*, and *Arisaematis rhizoma*. Pharmacological research highlights the various therapeutic properties of these components (given in Table 1). For example, *Trichosanthes kirilowii* extract has been shown to accelerate wound healing and possesses antibacterial and anti-inflammatory effects [20-22]. *Rhei radix et rhizoma* inhibits inflammation via NF-κB inactivation [23], and *Phellodendri cortex* has both anti-inflammatory and antibacterial properties [24,25]. Curcumin, the active component in *Curcumae longae rhizoma*, exhibits antioxidant, antimicrobial, and wound-healing effects through growth factor induction [26]. Studies on *Angelicae dahuricae radix* highlight its antinociceptive and anti-inflammatory activities [27]. *Magnoliae cortex*, rich in magnolol, is noted for its anti-inflammatory and antimicrobial activities [28-38]. *Glycyrrhizae radix et rhizoma* has shown anti-inflammatory effects through inhibition of PGE2, TXB2, and LTB4 [39], along with antimicrobial properties [40-42]. *Citri reticulatae pericarpium vetum* offers significant antioxidant and anti-inflammatory benefits [43-46]. *Atractylodis rhizoma* demonstrates antifungal, antibacterial, antioxidant, and anti-inflammatory activities [47-50], while *Arisaematis rhizoma* has been found to have anti-inflammatory and analgesic effects [51,52].

**Table 1:** The proportion of Ru-Yi-Jin-Huang-Saan (RYJHS) and the efficacy of its ingredients.

*Latin crude drug name* (English name)	Plant part	Proportion	Efficacy
*Trichosanthis radix* (Trichosanthes root)	Root	25%	Wound healing, antibacterial, and anti-inflammatory effects
*Rhei radix et rhizoma *(Rhubarb)	Root and rhizome	12.5%	Anti-inflammatory effect
*Phellodendri cortex* (Phellodendron bark)	Bark	12.5%	Antibacterial and anti-inflammatory effects
*Curcumae longae rhizoma* (Turmeric rhizome)	Rhizome	12.5%	Wound healing, antioxidant, radical scavenging, antimicrobial, and anti-inflammatory effects
*Angelicae dahuricae radix* (Dahurian Angelica root)	Root	12.5%	Antinociceptive and anti-inflammatory effects
*Magnoliae cortex* (Magnolia bark)	Bark	5%	Anti-inflammatory and antimicrobial effects
*Glycyrrhizae radix et rhizoma* (Liquorice root and rhizome)	Root and rhizome	5%	Anti-inflammatory and antimicrobial effects
*Citri reticulatae pericarpium vetum* (Aged tangerine peel)	Peel	5%	Antioxidant and anti-inflammatory effects
*Atractylodis rhizoma* (Atractylodes rhizome)	Rhizome	5%	Antifungal, antibacterial, antioxidant, and anti-inflammatory effects
*Arisaematis rhizoma* (Jackinthepulpit tuber)	Rhizome	5%	Anti-inflammatory and analgesic effects

Further research indicates that RYJHS can alleviate inflammatory pain without causing sensitization [53]. Clinically, it is used to manage conditions such as phlebitis [54], osteoarthritis of the knee [55], gout, diabetic foot ulcers [56], and herpes zoster.

However, despite the numerous studies mentioned above, there is still a lack of clinical research on RYJHS. This study aims to evaluate the efficacy and adverse effects of using RYJHS on Colles fracture after surgery.

## Methods

### Study Design

The study is a randomized, double-blind, placebo-controlled trial design based on SPIRIT (Standard Protocol Items: Recommendations for Interventional Trials) reporting guidelines (Multimedia Appendix 1) [57], and the results will follow the CONSORT (Consolidated Standards of Reporting Trials) guidelines [58]. Our research project is scheduled to recruit patients from May 1, 2023, to April 30, 2025. All postoperative patients with Colles fracture will be recruited through referrals from orthopedic physicians at Changhua Christian Hospital. Researchers will screen and select participants based on specific inclusion and exclusion criteria. In this study, all participants are randomly assigned using a computerized block randomization schedule. We randomly generate a pool of 100 patients, who are assigned to sequentially numbered opaque envelopes. The treatment allocations are balanced within each group, with each group containing 50 patients. Researchers and patients are both blinded to the treatment allocation, with the exception of the statistician responsible for the randomization process. The two groups in the study consisted of the experimental group receiving RYJHS treatment and the control group receiving a placebo.

### Study Settings and Participants

**Figure 1:**
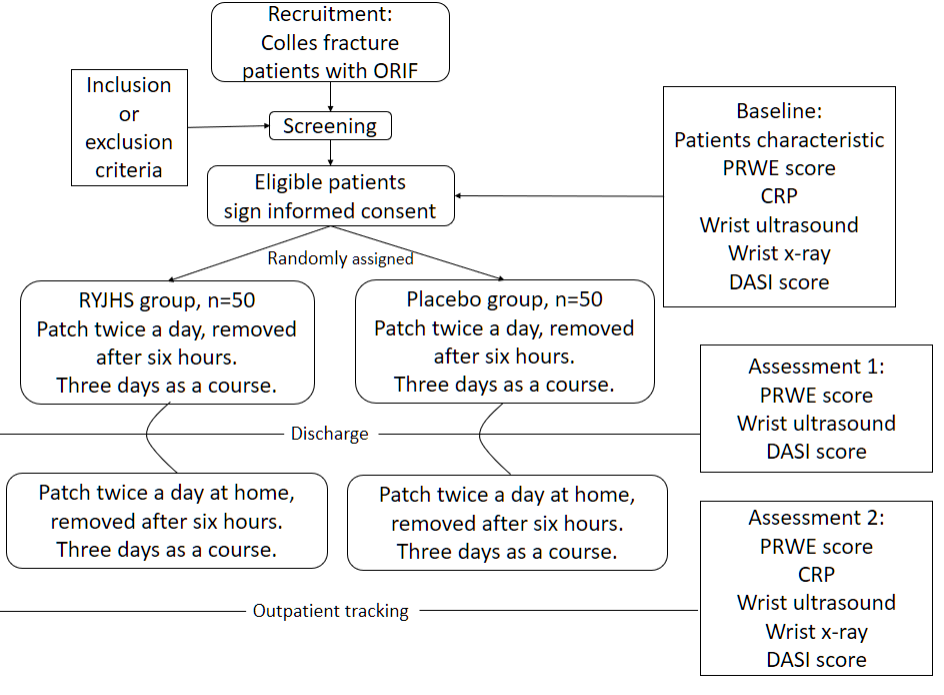
CONSORT (Consolidated Standards of Reporting Trials) flow diagram of enrollment, randomization, treatment, and evaluation. CRP: C-reactive protein; DASI: Dyshidrotic Eczema Area and Severity Index; ORIF: open reduction and internal fixation; PRWE: Patient-Rated Wrist Evaluation; RYJHS: Ru-Yi-Jin-Huang-Saan.

**Figure 2:**
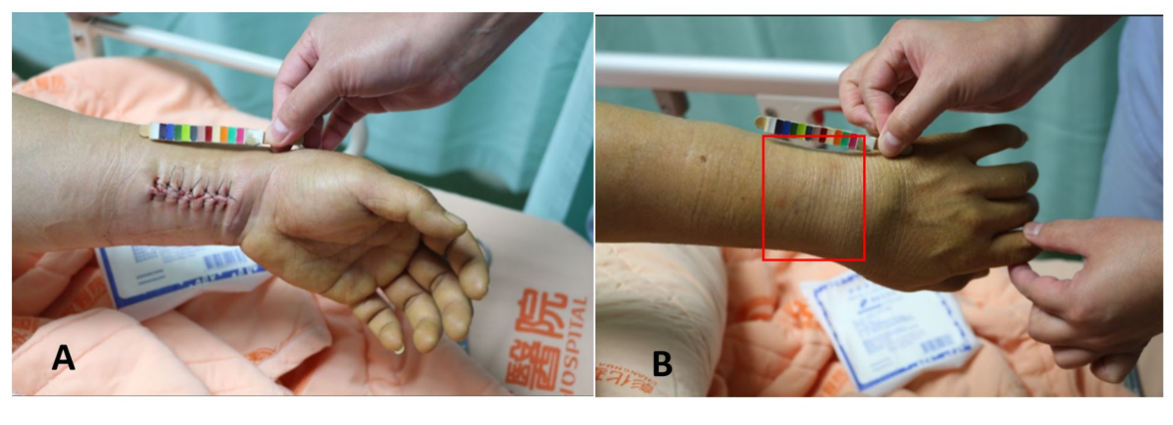
Applying Ru-Yi-Jin-Huang-Saan (RYJHS) plaster on Colles fracture after surgery. (A) The surgical incision site. (B) The site of the RYJHS plaster on a Colles fracture after surgery.

All participants undergo standard medical treatment after surgery and are involved in the efficacy assessment of RYJHS external application. Figure 1 shows the study’s flowchart. Both groups apply a patch plaster on the back of the wrist without wounds (avoiding the suture of the fracture operation). Figure 2 shows the site of the medication application. The experiment group apply the RYJHS plaster, while the control group apply a placebo plaster. The patch is applied twice a day, for 6 hours each time, with a 6-hour break in between, repeated for 3 days, completing one course of treatment. Patients undergo two courses of treatment, with the first course completed during hospitalization, and the second course completed 3 days after discharge.

### Inclusion Criteria

To be eligible for our study, patients must meet the following criteria: be 20-80 years of age, have a Colles fracture (Frykman classification type I-VI) [59] diagnosis that has been treated with open reduction and internal fixation (ORIF) surgery, and provide informed consent either personally or through their family members.

### Exclusion Criteria

The following exclusion criteria will be applied: age older than 80 years or younger than 20 years; inability to comply with experimental procedures or complete questionnaires; presence of wounds on the back of the wrist; allergy to the herbal patch before; use of other Chinese herbal topical medicine after fracture; pregnancy; cancer; stroke; and systemic diseases such as severe anemia, thyroid disease, and poorly controlled diabetes.

### Sample Size Calculation

Our study closely resembles the design of phase-2 studies in clinical trials, which aim to assess the effectiveness of drugs in participants with specific conditions or diseases. We used G*Power (Heinrich-Heine-Universität Düsseldorf) to estimate the necessary sample size for our study, taking into account repeated-measures ANOVA within-between interactions with a medium effect size of f=0.25 and α level=.05. To achieve a statistical power of 0.95, we calculated a total sample size of 86 [60]. To account for potential dropouts and satisfy our inclusion criteria, we will enroll 100 individuals who have been admitted to our orthopedic care ward and diagnosed with Colles fracture after surgery.

### Study Medication

**Figure 3:**
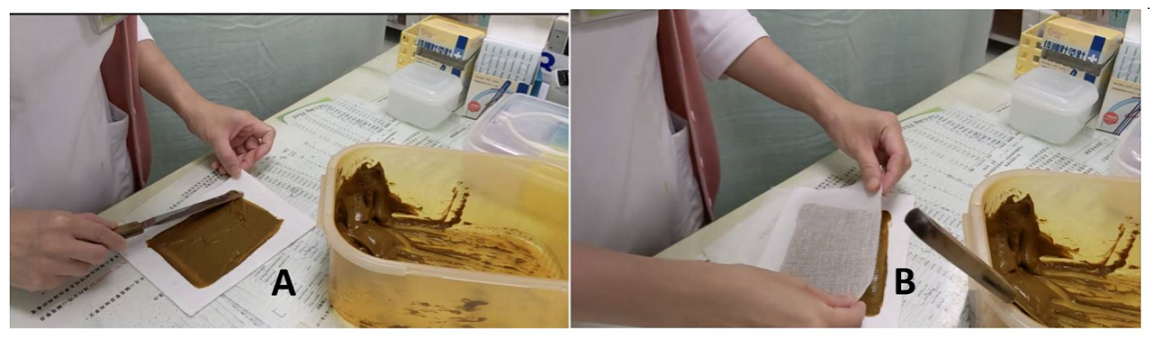
Preparation of Ru-Yi-Jin-Huang-Saan (RYJHS) plaster. (A) Spread the paste onto a cotton cloth and (B) cover with gauze.

RYJHS is a common TCM plaster that has been used for more than 500 years. RYJHS is composed of 10 herbs: *Trichosanthis radix*, *Rhei radix et rhizoma*, *Phellodendri cortex*, *Curcumae longae rhizoma*, *Angelicae dahuricae radix*, *Magnolite cortex*, *Glycyrrhizae radix et rhizoma*, *Citri reticulatae pericarpium vetum*, *Atractylodis rhizoma*, and *Arisaematis rhizoma* (given in Table 1). This herbal formula is a fixed prescription announced by the Department of Chinese Medicine and Pharmacy. The RYJHS used in our study is manufactured by Kaiser Pharmaceutical Co and meets the requirements of Good Manufacturing Practice. It has also been issued a drug certificate. The placebo powder, which uses computer color-matching technology in the color simulation of RYJHS [61], is also produced by Kaiser Pharmaceutical Co. Both RYJHS and the placebo are prepared by mixing 13 grams of powder with 23 mL of water, which is then evenly spread onto a cotton cloth and covered with gauze for later use. The aforementioned procedures are all carried out by the same experienced technician, as given in Figure 3.

### Outcome Measurements

In this study, we will gather data from each participant including their gender, age, affected hand and dominant hand, Patient-Rated Wrist Evaluation (PRWE) score, C-reactive protein (CRP) levels, wrist ultrasound results, wrist x-ray images, and Dyshidrotic Eczema Area and Severity Index (DASI) score. Patients who have undergone Colles fracture surgery are required to be evaluated, and data will be collected before the trial, as well as 3 days and 6 days after topical medication application. The participant timetable of enrollment, assessments, and treatments is given in Table 2. The primary outcome is the PRWE score, which is used to assess wrist function recovery. The PRWE score, collected via a questionnaire, was developed in Canada for patients with wrist problems to express their pain and level of function [62]. The secondary outcomes are CRP level and wrist imaging records, which are used to demonstrate the degree of inflammation and swelling reduction. In addition, the DASI score is used to monitor any allergic or adverse events that may occur at the application site [63].

**Table 2:** Timetable of enrollment and assessments.

Time			Screening		Baseline		Treatment 1 at hospital		Treatment 2 at home
			D_0_^a^		D_1_		D_3_		D_6_
**Enrollment**									
	Inclusion or exclusion	✓		—^b^		—		—	
	Informed consent	✓		—		—		—	
	Demographic data	✓		—		—		—	
	Dominant and affected hand	—		✓		—		—	
	Medical history	✓		—		—		—	
	Randomization	—		✓		—		—	
**Assessment**									
	PRWE^c^ score	—		✓		✓		✓	
	CRP^d^	—		✓		—		✓	
	Ultrasound	—		✓		✓		✓	
	X-ray	—		✓		—		✓	
	DASI^e^ score	—		✓		✓		✓	
	Adverse events	—		Record at any time^f^		Record at any time		Record at any time	

^a^D_x_: number of days into the experiment.

^b^Not available.

^c^PRWE: Patient-Rated Wrist Evaluation.

^d^CRP: C-reactive protein.

^e^DASI: Dyshidrotic Eczema Area and Severity Index.

^f^If a patient has any side effects during the experiment, they must be recorded immediately.

### Statistical Analysis

We will conduct statistical analyses using the SPSS software (version 22; IBM Corporation). Descriptive analyses will be performed on demographic data using frequencies and percentages to characterize the sample. Categorical variables will be compared using chi-square tests, while continuous variables will be compared using 2-tailed *t* tests. We will use repeated-measures ANOVA to determine if changes in wrist function (the dependent variable) are due to the interaction between the “type of treatment” (RYJHS) and “time” (measurement time). Multiple regression will be used to assess the impact of latent factors on primary outcomes (wrist function) and secondary outcomes (swelling and inflammation), adjusting for all possible covariates. Regression models will be performed in different outcome groups to compare the effects of different treatments (RYJHS intervention vs placebo).

### Ethical Considerations

Approval for this trial (protocol ID 221006) was granted by the Institutional Review Board of Changhua Christian Hospital (CCH) on November 25, 2022, following the principles outlined in the Declaration of Helsinki. The study protocol has also been registered on ClinicalTrials.gov (NCT05638360). Individuals interested in participating will be required to provide written informed consent before the study’s initiation. They will receive comprehensive information about the study, including procedures, potential benefits, and risks, excluding specific details regarding the RYJHS medication. Participants are free to withdraw at any time without any impact on their future medical care.

Data collected will be anonymized to protect participants’ privacy, and all information will be kept confidential according to institutional data protection policies. No direct compensation will be provided to participants. The results of this research will be disseminated through publication in a peer-reviewed journal and presented at scientific conferences.

## Results

The protocol was registered on ClinicalTrials.gov (NCT05638360) on December 6, 2022. Patient recruitment commenced in May 2023, with the first patient enrolled on June 15, 2023, and is projected to continue until April 30, 2025. As of December 2023, a total of 32 patients have been enrolled. Data analysis and report preparation are expected to be completed by the end of 2025.

## Discussion

### Expected Findings

The primary clinical treatments for Colles fractures, such as closed reduction, casting, percutaneous fixation, external fixation, and ORIF, generally yield positive functional outcomes. However, both percutaneous and external fixation have reported a higher risk of infection, while percutaneous fixation has been shown to have a higher rate of soft tissue injuries [64,65]. Besides, the ORIF procedure including local dissection, reduction, and the insertion of plates or screws results in a higher incidence of tendonitis, tendon irritation, or tendon rupture [66,67]. In Taiwan, the routine conventional treatment for Colles fracture is ORIF surgery. However, operations often result in soft-tissue and lymphatic vessel damage. As a result, patients may experience heat, pain, redness, and swelling after the operation. The discomfort disrupts their willingness to undergo rehabilitation. Prolonged swelling can also affect the range of motion, hand function, muscle strength, and outward appearance of these patients [68]. Therefore, resolving pain and swelling is a significant problem for patients with Colles fracture after ORIF surgery.

The most common method of relieving swelling is ice packing. Ice packing can reduce swelling, capillary permeability, and delivery of inflammatory substances [69]. Besides, ice packing can decrease nerve conduction velocity, increase pain threshold, and provide analgesia [70]. However, many studies have reported that ice packing not only delays wound healing but also has a higher risk of cold injury [71,72]. Besides, pain management is also a concern for patients who undergo ORIF surgery. Physicians often prescribe opioids; NSAIDs; or a combination of opioids, NSAIDs, and steroids for pain management [73]. However, due to the potential side effects, these medications raise concerns for patients with diabetes, hypertension, gastrointestinal disorders, impaired liver function, or impaired kidney function.

There are several limitations in this study design. First, during the patients’ hospitalization, patients were assisted by nurses who ensured regular medication application. However, it may be challenging to confirm whether the patients continued to apply the medication on schedule after discharge. Therefore, it might be necessary to use phone reminders to engage caregivers in assisting with regular medication application. Second, theoretically, the closer the topical medication is applied to the lesion, the more effective it is likely to be. However, due to concerns about postsurgical wound infections, the application of topical medication needs to be avoided on the site of surgical incision. Therefore, for the purpose of this experiment, only the uninjured area on the back of the wrist can be selected for topical medication application. Finally, the inclusion criteria for the trial of Colles fracture did not encompass comminuted fractures; thus, the efficacy of RYJHS may not be evaluated in patients with severe Colles fractures.

This study represents the first randomized, double-blind, placebo-controlled trial to investigate the efficacy of RYJHS for postoperative Colles fractures. RYJHS, with its natural ingredients known for anti-inflammatory and antibacterial properties, could serve as a noninvasive adjunctive therapy to reduce postoperative complications and enhance functional recovery. Its application aligns with patients’ increasing interest in integrative medicine options that minimize reliance on pharmaceuticals. Future directions include exploring RYJHS’s active ingredients, assessing formulation efficiency, and developing a more accessible application method, which may strengthen RYJHS’s role as an adjunctive treatment for Colles fractures.

### Conclusion

This randomized, double-blind, placebo-controlled trial aims to provide robust evidence of the efficacy and safety of RYJHS as a topical adjunctive treatment for postsurgical Colles fractures. With the potential to reduce inflammation and aid functional recovery, RYJHS could offer an alternative, nonpharmaceutical option for managing postoperative complications in patients with Colles fractures.

## Appendix

Multimedia Appendix 1 SPIRIT (Standard Protocol Items: Recommendations for Interventional Trials) checklist.

## Abbreviations

CRP: C-reactive protein
CONSORT: Consolidated Standards of Reporting Trials
DASI: Dyshidrotic Eczema Area and Severity Index
NSAID: nonsteroidal anti-inflammatory drug
ORIF: open reduction and internal fixation
PRWE: Patient-Rated Wrist Evaluation
RYJHS: Ru-Yi-Jin-Huang-Saan
SPIRIT: Standard Protocol Items: Recommendations for Interventional Trials
TCM: traditional Chinese medicine

## Data Availability

Since this protocol does not contain any data, it will be provided after analysis and published in a journal upon trial completion. Data can be made available upon the author’s request.
